# Effect of Magnesium Incorporation on Solution-Processed Kesterite Solar Cells

**DOI:** 10.3389/fchem.2018.00005

**Published:** 2018-01-26

**Authors:** Raquel Caballero, Stefan G. Haass, Christian Andres, Laia Arques, Florian Oliva, Victor Izquierdo-Roca, Yaroslav E. Romanyuk

**Affiliations:** ^1^Departamento de Física Aplicada, Universidad Autónoma de Madrid, Madrid, Spain; ^2^Laboratory for Thin Films and Photovoltaics, Empa- Swiss Federal Laboratories for Materials Science and Technology, Dübendorf, Switzerland; ^3^Catalonia Institute for Energy Research (IREC), Barcelona, Spain

**Keywords:** thin film solar cells, kesterite, Mg, solution processing, structural defects

## Abstract

The introduction of the alkaline-earth element Magnesium (Mg) into Cu_2_ZnSn(S,Se)_4_ (CTZSSe) is explored in view of potential photovoltaic applications. Cu_2_Zn_1−x_Mg_x_Sn(S,Se)_4_ absorber layers with variable Mg content *x* = 0…1 are deposited using the solution approach with dimethyl sulfoxide solvent followed by annealing in selenium atmosphere. For heavy Mg alloying with *x* = 0.55…1 the phase separation into Cu_2_SnSe_3_, MgSe_2_, MgSe and SnSe_2_ occurs in agreement with literature predictions. A lower Mg content of *x* = 0.04 results in the kesterite phase as confirmed by XRD and Raman spectroscopy. A photoluminescence maximum is red-shifted by 0.02 eV as compared to the band-gap and a carrier concentration N_CV_ of 1 × 10^16^ cm^−3^ is measured for a Mg-containing kesterite solar cell device. Raman spectroscopy indicates that structural defects can be reduced in Mg-containing absorbers as compared to the Mg-free reference samples, however the best device efficiency of 7.2% for a Mg-containing cell measured in this study is lower than those frequently reported for the conventional Na doping.

## Introduction

Kesterite-type material Cu_2_ZnSn(S,Se)_4_ (CZTSSe) has been recognized as a promising candidate for low-cost thin-film solar cells due to its large absorption coefficient, tunable band-gap E_g_ between 1.0 and 1.5 eV adjusted via S/Se-ratio, low toxicity and earth-abundant nature. This technology has achieved a 12.6% maximum performance (Wang C. et al., 2014), still far away from that of 22.6% for Cu(In,Ga)Se_2_ solar cells (Jackson et al., 2016). The main performance limitation of kesterite-based solar cells is the open circuit voltage deficit (E_g_/q − V_oc_). One of the reasons is the non-optimal quality of kesterite absorber and the presence of secondary phases (Siebentritt and Schorr, 2012). Another reason can be the unfavorable alignment of the conduction band minimum (CBM) at the CZTSSe/CdS interface (Platzer-Björkman et al., 2015). Gokmen et al. (2013) pointed out that the [Cu_Zn_ + Zn_Cu_] defect cluster could be the origin of electrostatic potential fluctuation in the CZTSSe absorber layer. That fluctuation is probably a significant factor that decreases the photovoltaic (PV) device performance. It was suggested that the substitution of Cu or Zn by other elements as Magnesium (Mg) could suppress the antisite defects Cu_Zn_ and/or Zn_Cu_ formation that limit kesterite solar cells efficiency (Zhong et al., 2016).

Several studies about the Mg incorporation into kesterite have recently been reported, but the observed effects of Mg are contradictory. CIGSe bulk material doped with Mg was deposited by liquid phase sintering method measuring a decreased hole concentration with the increase in Mg content (10 at %, Mg/(In+Mg) = 0.1), which was attributed to the Mg_Cu_ donor defect (Monsefi and Kuo, 2014). At the same time, an increased hole concentration was observed for 5 at % Mg explained by the substitution of In with Mg ion to form Mg_In_ acceptor defect. Cu_2_MgSnS_4_ thin films grown by ultrasonic co-spray pyrolysis showed p-type conductivity and band-gap energy of 1.76 eV (Guo et al., 2016). In contrary, n-type conductivity was estimated for (Cu_2_–_x_Mg_x_)ZnSnSe_4_ bulk materials with *x* = 0.1−0.4, which was attributed to the formation of the donor-type Mg_Cu_ antisite defects (Kuo and Wubet, 2014). The formation of stable Cu_2_MgSn(S,Se)_4_ was calculated based on density functional theory (Zhong et al., 2016), whereas a complete phase separation was predicted by Wang W. et al. (2014).

The purpose of this work is to study the effect of Mg addition in various concentrations to CZTSSe solar cell absorbers. Mg is incorporated to the absorber thin films by adding a magnesium salt to the precursor solution. Two experimental series are presented. The first experiment involves the replacement of Zn for Mg concentrations in order to evaluate possible alloying effects on the absorber band-gap for Cu_2_Zn_1−x_Mg_x_Sn(S,Se)_4_ thin films, however a complete phase separation is observed for *x* = 0.55 and 1. For a lower Mg content of *x* = 0.04 the kesterite phase is obtained, and respective absorbers and solar cells are studied to reveal any structural and electronic effects of Mg-containing sample as compared to a nominally Mg-free one.

## Materials and methods

### Absorber preparation

The Cu_2_Zn_1−x_Mg_x_Sn(S,Se)_4_ thin films absorbers were deposited from the precursor solution with dimethyl sulfoxide (DMSO) as the solvent onto Mo/SiO_x_/soda-lime glass (SLG) substrates with a subsequent selenization using the methodology described in the previous work (Haass et al., 2015). 1 mm-thick SLG was cleaned in three different supersonic baths at a temperature of 80°C. The first bath consisted of salt-free water with soap (Borer Deconex), the second bath consisted of a weak acetic solution (~5%) and the final bath contained only de-ionized water (18 MΩ·cm). The residual water was blown off with nitrogen and the substrates were dried in vacuum prior to the subsequent layer deposition. The SiO_x_ barrier layer was deposited at 200°C substrate temperature on SLG substrate by sputtering of a Si target in an Ar/O_2_ atmosphere. The layer thickness of SiO_x_ is approximately 200-300 nm. The SiO_x_ barrier layer was used to reduce the alkali elements out-diffusion from the SLG substrate although the presence of Na could not be eliminated since it can also be transported via the gas phase during the annealing step (Abzieher et al., 2016). The CZTSSe precursor solution contained thiourea (99%+, Sigma-Aldrich), SnCl_2_·2H_2_O (98%, Sigma-Aldrich), ZnCl_2_ (99.99%, Alfa Aesar) and CuCl_2_ (98%+, Alfa Aesar) dissolved in DMSO (99.9%, Alfa Aesar). Mg was introduced by adding Mg(CH_3_COO)_2_·2H_2_O to the precursor solution. Table 1 shows the nominal Mg composition and metal ratios in the precursor solutions.

**Table 1:** Mg content and nominal metal ratios in the precursor solution (sol) and selenized absorbers (abs) for all the samples.

**Sample**	**Nominal Mg content x in Cu_2_Zn_1−x_Mg_x_Sn(S,Se)_4_ in the precursor solution**	Nominal metal ratios in precursor solution (sol) and metal ratios measured by ICPMS and/or XRF^*^ **in selenized absorbers (abs)**			**Actual Mg content measured by ICPMS in selenized absorbers**
		**Cu/Zn**	**Cu/Sn**	**Zn/Sn**	
A	*x* = 1.00	N.A.	1.08 (sol)	0 (sol)	*x* = 1.00
		N.A.	1.19 (abs)	0 (abs)	
B	*x* = 0.50	2.46 (sol)	1.08 (sol)	0.44 (sol)	*x* = 0.55
		2.72 (abs)	0.99 (abs)	0.36 (abs)	
C	*x* = 0.17	1.28 (sol)	1.34 (sol)	1.05 (sol)	*x* = 0.04
		1.45 (abs)	1.74 (abs)	1.20 (abs)	
		1.41^*^(abs)	1.82^*^(abs)	1.29^*^(abs)	
D	*x* = 0.00	1.27 (sol)	1.40 (sol)	1.05 (sol)	*x* = 0.00
		1.40^*^(abs)	1.74^*^(abs)	1.24^*^(abs)	

* *Measurements carried out by XRF*.

The solution was spin-coated onto a Mo/SiO_x_-coated and dried on a hotplate at 320°C in air. The spin-coating and drying steps were repeated 12 times in order to obtain the desired precursor film thickness of approximately 1.5–2 μm (Haass et al., 2015). Samples were annealed in a rapid thermal annealing (RTP) reactor (AS-ONE 150 from Annealsys) inside a silica-coated graphite box with selenium pellets (0.8 g). A three- stage temperature gradient was employed for annealing samples with holding temperatures at 300, 500, and 550°C for 30, 45, and 5 min, respectively. After selenization the absorbers were immersed into a 10 wt% KCN solution for 30 s in order to remove any copper-rich phases and clean the surface from contaminations and oxides (Haass et al., 2015).

### Device preparation

A 50–70 nm thick CdS buffer layer was deposited by chemical bath deposition, and 70 nm/250 nm i-ZnO/Al:ZnO bilayer was sputtered. A Ni/Al top grid and an antireflection coating of MgF_2_ were deposited by e-beam evaporation. Individual solar cells were mechanically scribed to an area of 0.30 ± 0.02 cm^2^ (Haass et al., 2015).

### Materials characterization

Inductively coupled plasma mass spectrometry (ICP-MS) measurements were carried out to determine the Mg content in the selenized absorber layers, which is listed in Table 1. The ICP-MS measurements were done on the full cells (without antireflection coating) for samples A and B with prior etching of the window layer by 5% acetic acid for 60 s and rinsing in distilled H_2_O. For the sample C with much lower Mg concentration we used the ICP-MS results of the absorber layer on SLG (no Mo) without any etching. The Mg content measured by ICP-MS for sample C is 0.04, which is lower than the nominal content of 0.17, indicating that Mg is lost during the absorber fabrication, and the reason needs to be investigated. Secondary Ion Mass Spectrometry (SIMS) measurements were recorded on a TOF-SIMS system from ION-TOF using O^2+^ primary ions with 2 keV of ion energy, a current of 400 nA and a raster size of 400 × 400 μm^2^. An area of 100 × 100 μm^2^ in the case of depth profiles was analyzed using Bi^+^ ions with 25 keV of ion energy. Energy dispersive X-ray (EDX) analysis and X-ray fluorescence (XRF) were used to quantify the composition of matrix elements. The XRF measurements were done on selenized absorber layers without any etching. Scanning Electron Microscopy (SEM) and EDX measurements were done on a Hitachi S-4800 electron miscroscope. X-ray diffraction (XRD) patterns were recorded in 2θ\θ scan mode using a Bruker D8 diffractometer with CuKα radiation (λ = 1.5418 Å̀, beam voltage: 40 kV, beam current: 40 mA, calibrated using Si100 and Si111 single crystals), a step size of 0.04° and a scan rate of 0.5 s/step (Haass et al., 2015). Grazing incidence XRD (GIXRD) measurements were collected with a PaNAlytical X'Pert Pro MPD diffractometer using Cu K_α_ radiation (λ = 1.5418 Å̀, beam voltage: 40 kV, beam current: 40 mA), a step size of 0.02°, a scan rate of 2 s/step and a multilayer mirror (Caballero et al., 2015). Detector scans with incident angles of 1°, 3°, and 5° were carried out. Raman scattering measurements were performed in back scattering configuration using a highly sensitive Raman apparatus developed at IREC consisting in a Horiba Jobin Yvon iRH320 spectrometer coupled with a low noise CCD detector cooled at −70°C. In this system, excitation and light collection were made through a macro optic system with a laser spot diameter of about 70 μm. Back-scattering measurements were performed under 633 and 785 nm excitation wavelengths by focusing laser spot directly onto the layer surface which allows the assessment of the absorber layer without any contribution from the upper layers. In the case of back surface measurements, laser spot was directly focused on the back surface after mechanical lift-off process. Excitation power was kept below 26 W/cm^2^ in order to avoid presence of thermal effects in spectra. The first-order Raman spectrum of monocrystalline silicon (Si) was measured as a reference before and after each Raman spectrum acquisition, and spectra were corrected by imposing Si first order at 520 cm^−1^ (Oliva et al., 2017).

### Device characterization

The *J-V* characterization of solar cells was performed under standard test conditions (100 mWcm^−2^, 25°C, AM1.5G) using a solar simulator calibrated with a certified Si diode. External Quantum Efficiency (EQE) spectra were recorded using a chopped white light source (900 W halogen lamp) with a LOT MSH-300 monochromator, which was calibrated with Si and Ge photodiodes. The illuminated area on the sample was 0.1 cm^2^ including grid lines. Photoluminescence (PL) spectra were measured on a FT300 fluorescence lifetime spectrometer from PicoQuant with a 639 nm pulsed diode laser as excitation source (pulse width 90 ps, repetition rate 10 MHz) and a thermoelectric cooled Hamamatsu NIR-PMT module H10330A-45 (rise time 0.9 ns, transit time spread 0.4 ns). Capacitance-Voltage (C-V) room temperature measurements were carried out with a LCR-meter from Agilent (E4990A) with an AC-voltage of 30 mV (Haass et al., 2015). JV-T was carried out in the temperature range from 138 to 298 K. For temperature dependent current-voltage measurements the solar cell was placed on temperature controlled Cu stage inside an evacuated cryostat cooled with liquid nitrogen and illuminated by a 900 W halogen lamp. The sample temperature was measured by thermocouples and regulated by a PID controller. The intensity of the incident light was varied by 2 orders of magnitude from approximately 1 - 140 mWcm^−2^ using neutral density filters.

## Results and discussion

### Alloying with high concentration of Mg: Cu_2_Zn_1−x_Mg_x_Sn(S,Se)_4_

**Figure 1:**
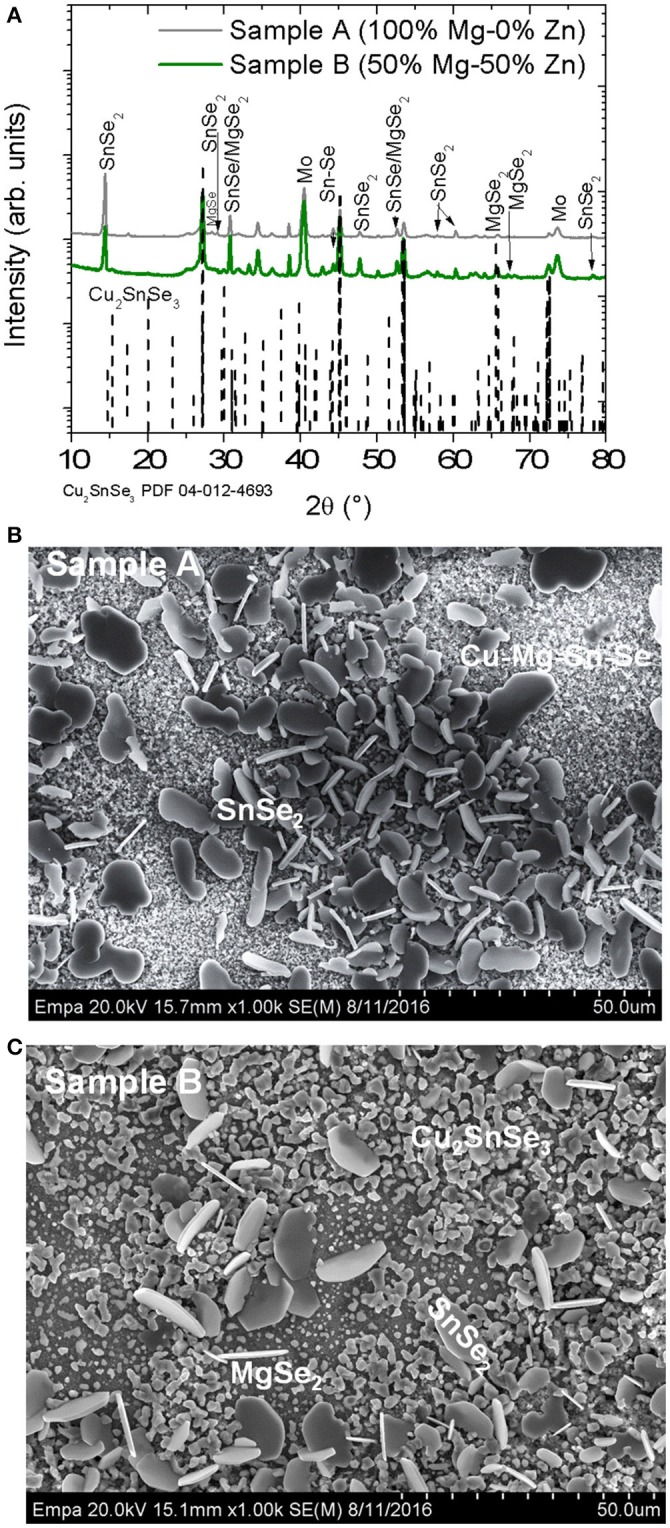
**(A)** GIXRD patterns of samples A and B exhibiting reflexes of Mo, Cu_2_SnSe_3_, SnSe, SnSe_2_, MgSe, and MgSe_2_. The PDF files number 04-012-4693, 04-009-2277, 01-089-2939, 04-018-3911, and 04-004-2933 have been used to identify Cu_2_SnSe_3_, SnSe, SnSe_2_, MgSe, and MgSe_2_ phases respectively. The dashed lines correspond to Cu_2_SnS_3_ phase. **(B,C)** Surface morphology of the samples A and B respectively. Different grains are formed, which are characteristic of the different secondary phases identified by EDX.

The first experiment series involved heavy alloying of Zn with Mg in Cu_2_Zn_1−x_Mg_x_Sn(S,Se)_4_ (CZMTSSe) thin films. Figure 1A shows XRD spectra of samples A (*x* = 1) and B (*x* = 0.5). One can observe a complete phase separation, identifying the reflexes corresponding to MgSe_2_, MgSe, SnSe_2_, and Cu_2_SnSe_3_ in both samples. The presence of CZTSSe and ZnSe phase is difficult to exclude due to the closeness of their diffraction peaks with those of Cu_2_SnSe_3_. Already during the deposition of the Cu_2_Zn_1−x_Mg_x_SnS_4_ (CZMTS) precursor layer, it was observed that the solution was inhomogeneous with some precipitates.

Figures 1B,C illustrate SEM surface morphology of samples A and B. Different types of grains are observed. For sample A (*x* = 1), SnSe_2_ is observed on top of smaller grains with the presence of Cu (15.1 at %)-Mg (16.9 at %)-Sn (10.1%)-Se (44.7 at%)-S (13.2 at %) (see Figure 1B). For sample B with *x* = 0.5, larger grains corresponding to SnSe_2_ and the smallest grains consistent with MgSe_2_ and Cu_2_SnSe_3_ are detected as shown in Figure 1C.

The observed phase separation is in agreement with the predictions of Wang et al. who describes the instability of the Cu_2_MgSnS_4_ by the following reaction:

(1) $$\begin{array}{r} \text{C}{\text{u}}_{2}\text{MgSn}{\text{S}}_{4}\to \text{MgS}+\text{C}{\text{u}}_{2}\text{Sn}{\text{S}}_{3} \end{array}$$

The calculated energy change of this reaction is exothermic (ΔE = −0.01 eV < 0), meaning that the phase separation of Cu_2_MgSnS_4_ proceeds spontaneously, in accordance with the disappearance of the stable region in the chemical potential space. The instability for this compound is directly related to the fact that for group IIA element, Cu_2_MgSnS_4_ is more stable in the ionic rocksalt structure with S than in the more covalent tetrahedral environment as in kesterite. When an I_2_-II–IV–VI_4_ compound is unstable, the corresponding elements, in this case Mg, can only be incorporated into a stable I_2_-II–IV–VI_4_ with low concentration (Wang W. et al., 2014). No working solar cells could be obtained using samples A and B as a consequence of the phase separation.

### Effect of low Mg concentration on kesterite solar cells

**Figure 2:**
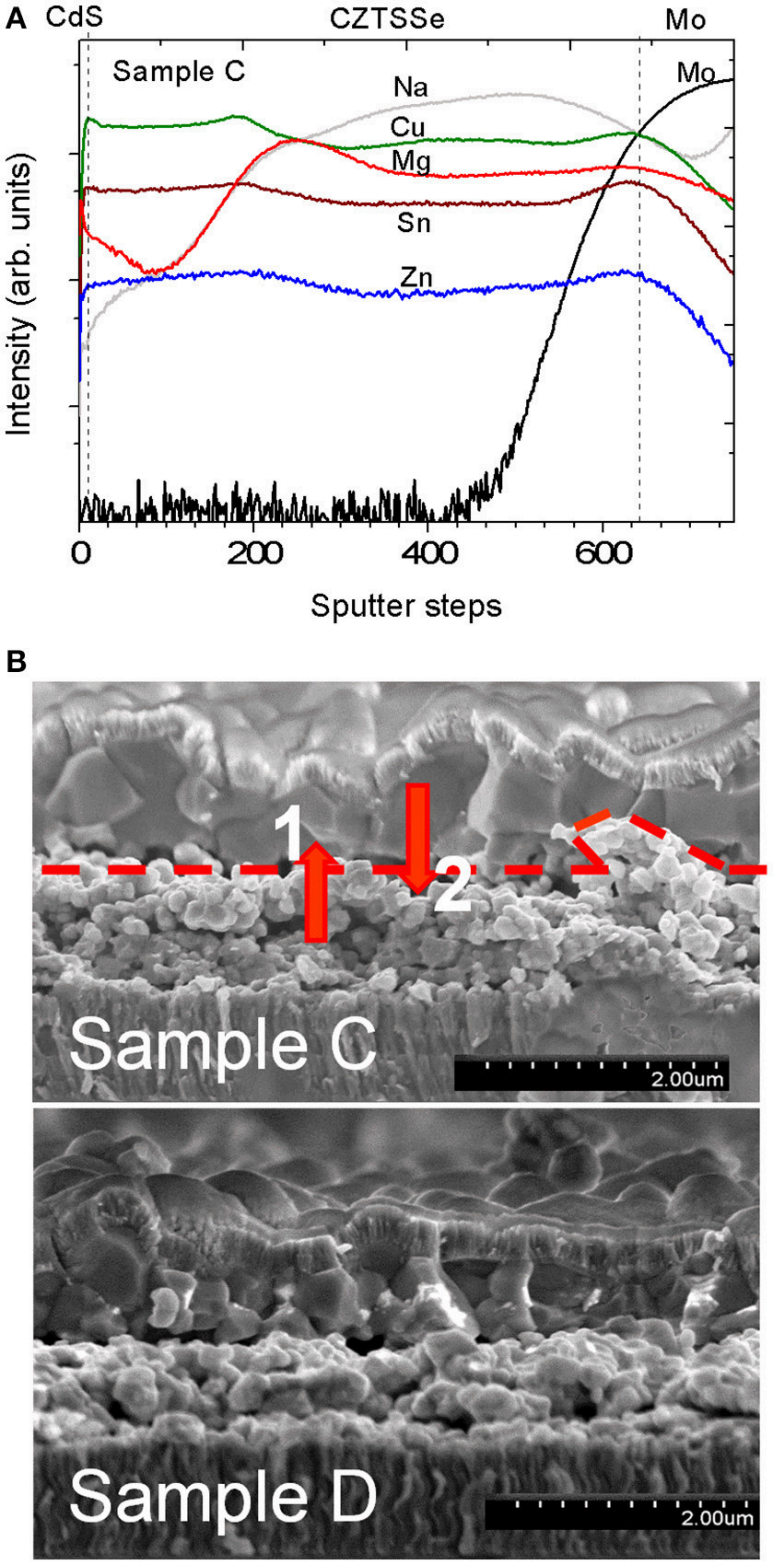
**(A)** SIMS depth profile of CdS/CZTSSe/Mo corresponding to device C. **(B)** Cross-sectional SEM micrographs of the completed devices C and D. In the device C the positions 1 and 2 correspond to the penetration depths where Raman measurements were carried out (see Figure 3). A dash line indicates the border where a clear change of grain size and crystallinity of the bilayer structure occur.

The effect of the addition of low Mg concentration was studied by comparing sample C with a Mg content of *x* = 0.04 (measured by ICP-MS in Table 1) to the nominally Mg-free, sample D. The distribution of Mg inside sample C measured by SIMS is not homogeneous (Figure 2A), with a higher Mg signal in the lower part of the absorber. This can be correlated to the morphology of samples C and D visible in the cross-section. Both absorbers exhibit a bi-layer structure with large-grain material on top and smaller grains close to the back contact as reported for the solution-processed kesterite layers (Haass et al., 2015). We assume that a higher Mg content in the lower part of sample C is due to a larger number of grain boundaries which can accommodate more Mg in comparison to large grains of the top crust. It is worth mentioning that a significant Na signal can be detected by SIMS in both samples C and D despite the fact that no Na was intentionally added to the precursor solution and a SiO_x_ barrier was employed. ICP-MS measurements were carried out to quantify the Na concentration incorporated into the absorber layer. A Mg-free reference sample had a sodium concentration of only 70 ppm, while samples without SiO_x_ barrier layer were reported with around 2,000 ppm (Sutter-Fella, 2014). This fact shows the effectiveness of the alkali barrier layer. This confirms that Na can also be transported via the gas phase during selenization (Abzieher et al., 2016).

**Figure 3:**
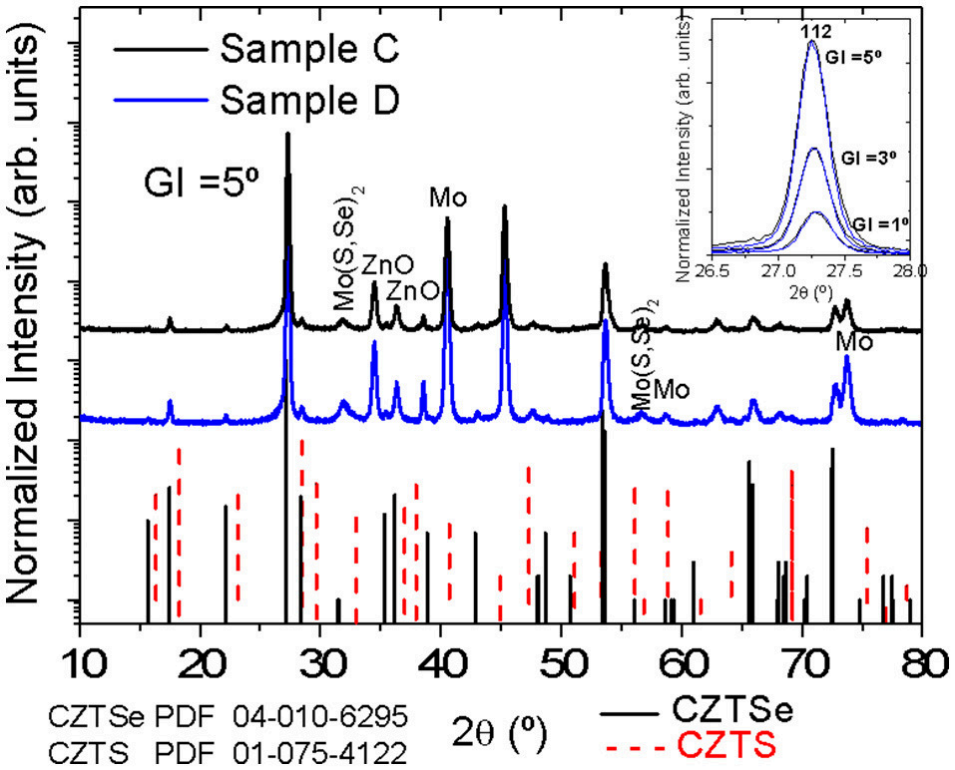
GIXRD patterns of samples C and D exhibiting reflexes of CZTSSe, Mo and Mo(S,Se)_2_. A zoom of the 112 Bragg peak measured at different grazing incidence angles do not show any significant difference between both samples. The PDF files number 04-010-6295 corresponding to CZTSe (solid line) and number 01-075-4122 assigned to CZTS (dash line) are also plotted.

Figure 3 displays GIXRD diffractograms of solar cells corresponding to samples C and D. Reflexes at 15.7°, 17.5° and 22.2° confirm unambiguously the CZTSSe phase. Moreover, reflexes at 31.8° and 56.6° appear, indicating the presence of the Mo(S,Se)_2_ layer formed during the selenization. No other secondary phases can be detected, although the presence of Zn(S,Se) and Cu_2_Sn(S,Se)_3_ phases cannot be ruled out since their diffraction reflexes coincide with those of CZTSSe. Reflexes of ZnO are also visible due to the window layer of i-ZnO/AZO in complete devices. A zoom of the 112 Bragg reflex of the kesterite phase measured at different grazing incidence angles does not show significant differences between both samples at different depths.

**Figure 4:**
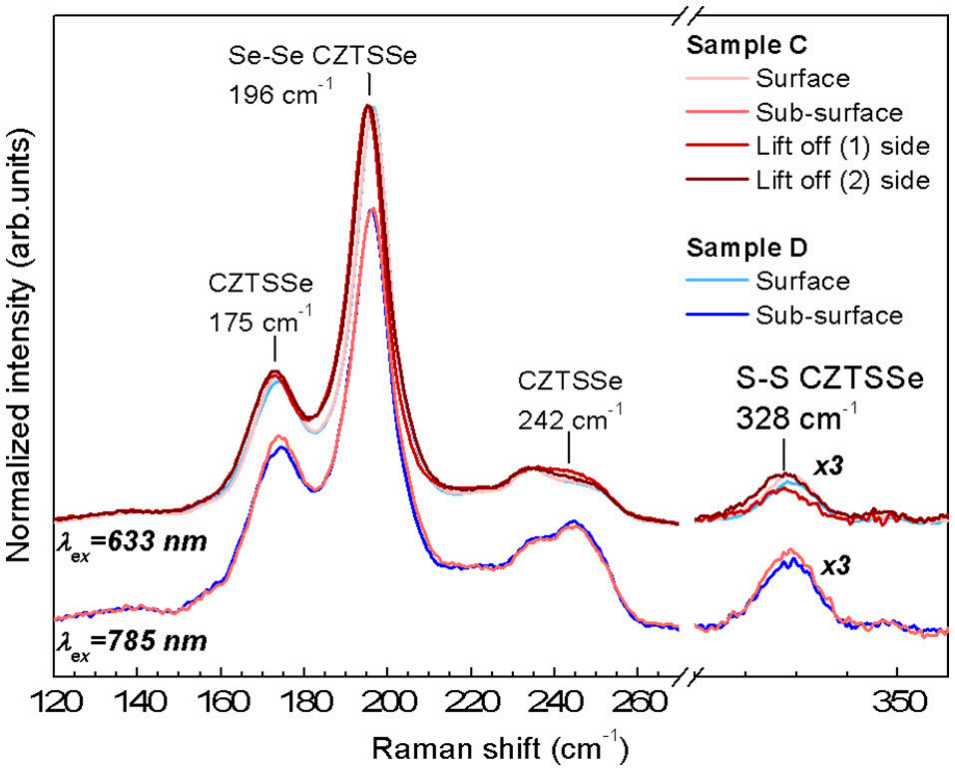
Raman spectra of the samples C and D acquired under 633 and 785 nm wavelengths (corresponding to different penetration depths, thus surface and sub-surface regions) and from the bulk of the absorber layer after lift-off process. (1) And (2) sides are the Raman spectra obtained at the regions (1) and (2) indicated in Figure 2B. The region centered at 330 cm^−1^ has been magnified x3 to clarify the contribution S-S vibration that indicates the S presence in the kesterite material.

Raman spectra measurements of the completed devices corresponding to samples C and D are shown in Figure 4 for two wavelengths of 633 and 785 nm. At these excitation wavelengths the contribution from the ZnO and CdS layers do not interfere with the absorber thin film signal allowing the direct characterization of the surface (penetration of < 70 nm for the 633 nm) and subsurface regions (< 150 nm for the 785 nm) (Dimitrievska et al., 2016b; Oliva et al., 2016). In order to access CZTSSe bulk, additional Raman measurements under 633 nm excitation were performed at the back of the Mg-containing CZTSSe after its mechanical delamination (lift-off process), corresponding to interface regions 1 and 2 (shown as red dotted line in Figure 2B). All Raman spectra exhibit characteristic features of highly Se-rich CZTSSe solid solution ([S]/([S]+[Se] ≈ 3% evaluated by Raman spectroscopy) yet. A detailed analysis of the Raman spectra of samples C and D can bring more insights about the impact of low Mg concentration on the CZTSSe absorber. Raman related parameters are summarized in Tables S1–S3 (See Supporting Information). The analysis of the Raman shift (RS) and the full-width-at-half-maximum (FWHM) of the Se-Se A' mode of kesterite phase (peak located at 196 cm^−1^) from Mg-containing/Mg-free samples at surface/sub-surface regions show that the differences are within the experimental error. The apparent FWHM increases between the surface and the sub-surface measurements (around 2 cm^−1^) (see Tables S1, S2 in Supporting Information) is attributed to the activation by Raman pre-resonant process of weak contributions overlapped with the 196 cm^−1^ peak. The absence of significant variations of the characteristic properties of the Se-Se peak suggests that both samples (Mg-containing/Mg-free) present a similar crystalline quality at the surface and sub-surface regions.

For the Mg-containing sample C an extended analysis has been performed to compare the surface/bulk and bulk regions. The results show a red-shift of the main peak as well as an increase of the FWHM. These changes are an indication of crystal quality degradation, in agreement with the smaller grains at the back of the absorber layer observed by cross-sectional SEM images (see Figure 2B). Additionally, this red-shift of the main peak could be compatible with the replacement of Cu or Zn by a lighter atom as Mg. As shown in Figure 2A, a minimum Cu SIMS-signal coincides with a maximum Mg SIMS-signal and is located near the border between the big and small grains shown in Figure 2B. These results seem to suggest that a replacement of Cu by Mg could take place in that region, which could reduce the Cu_Zn_ antisite defects formation.

**Figure 5:**
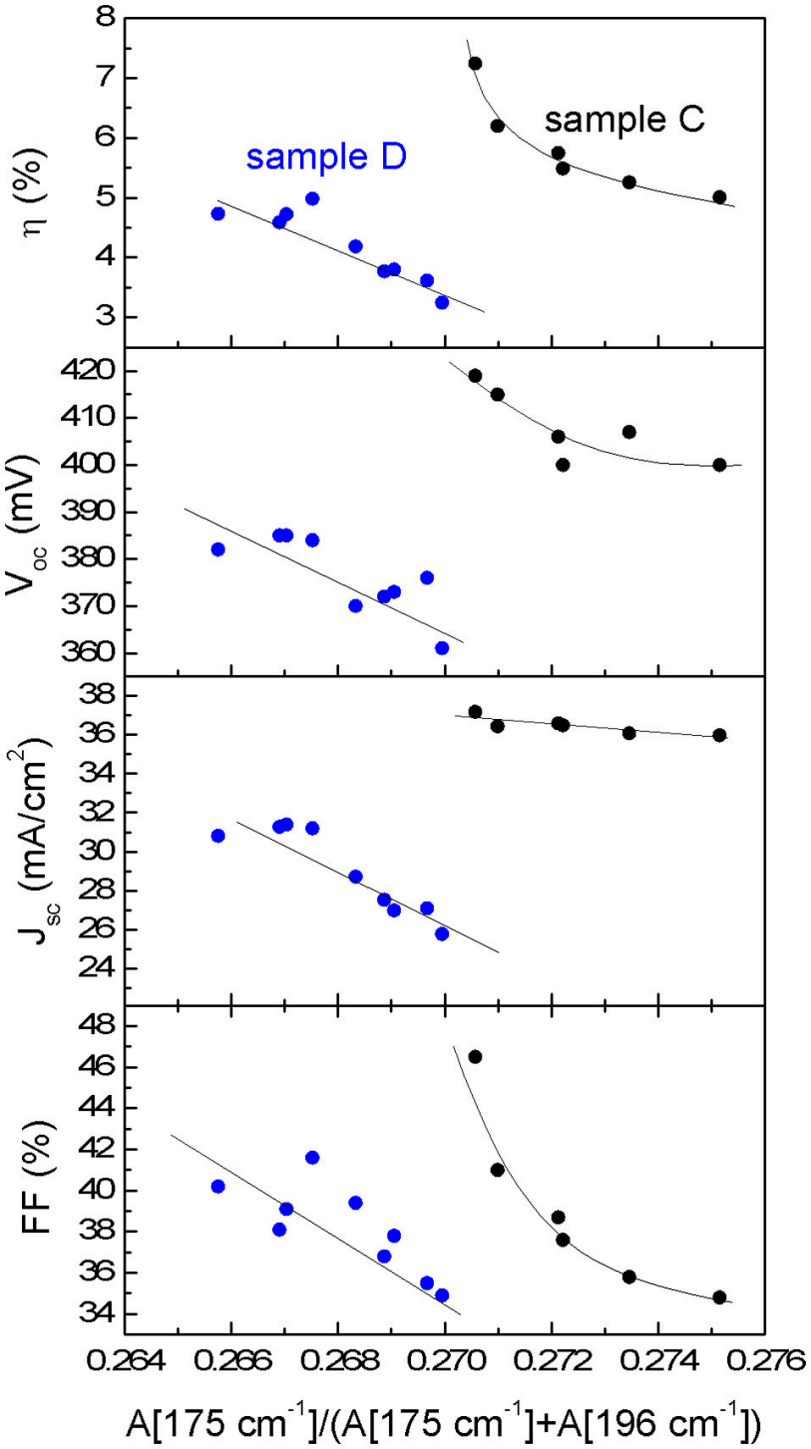
Correlation of the photovoltaic parameters of devices C and D with the A[175 cm^−1^]/(A[175 cm^−1^] + A[196 cm^−1^]) Raman intensity ratio.

The analysis of the changes at 175 and 242 cm^−1^ regions is interesting in order to evaluate the concentration variation of the defect clusters [Zn_Cu_+V_Cu_] and [2Zn_Cu_+Zn_Sn_] (Dimitrievska et al., 2015). A significant variation of the 175 cm^−1^ peak intensity between the samples C and D at the surface and the sub-surface is observed. The 175 and 196 cm^−1^ peak intensity ratio has been correlated with the concentration of the [Zn_Cu_+V_Cu_] (A-type) defect cluster and with the V_oc_ of the device (Dimitrievska et al., 2015). For the Mg-containing samples an increase of this ratio is observed. This suggests that with the inclusion of Mg, a reduction of V_cu_ and/or Zn_Cu_ antisite formation is promoted. It is in agreement with the hypothesis that for low Mg concentration in CZTSSe samples, Mg replaces Cu and/or the Mg allows the inhibition of Zn_Cu_ antisites formation during CZTSSe synthesis (Zhong et al., 2016). Additionally the analyses of the bulk region show similar values of the 175 cm^−1^ peak intensity than those observed for the surface indicating a similar defect property at the surface and bulk of the absorber layer. Furthermore, the variation of the PV parameters of both samples with the A[175 cm^−1^]/([A175 cm^−1^)+A[196 cm^−1^]) ratio (see Figure 5) shows a clear correlation between the improvement of V_oc_, short circuit current density J_sc_, fill factor FF and efficiency η with the increase of the [Zn_Cu_+V_Cu_] defect cluster concentration (reduction of the A[175 cm^−1^]/([A175 cm^−1^)+A[196 cm^−1^]) ratio), similarly as what has already been reported in previous works for pure CZTSe (Dimitrievska et al., 2016a,b). Therefore, optoelectronic parameters increase with the increase of the defect cluster concentrations as it is shown in Figure 5 for both samples, Mg-containing and Mg-free. This fact suggests that Mg improves material properties allowing better device performances, but reduces the [Zn_Cu_+V_Cu_] defect clusters density.

The evolution of the peak intensity at 242 cm^−1^ shows that for the Mg-containing and Mg-free samples, the surface and sub-surface regions are similar while a clear increase is observed for the spectra acquired from the points (1) and (2) of the lift-off for sample C, regions with smaller grain size. This suggests that for this region, the [2Zn_Cu_+Zn_Sn_] defect cluster is promoted inducing the degradation of the crystal quality observed by changes of the FWHM and Raman shift (Dimitrievska et al., 2015).

**Figure 6:**
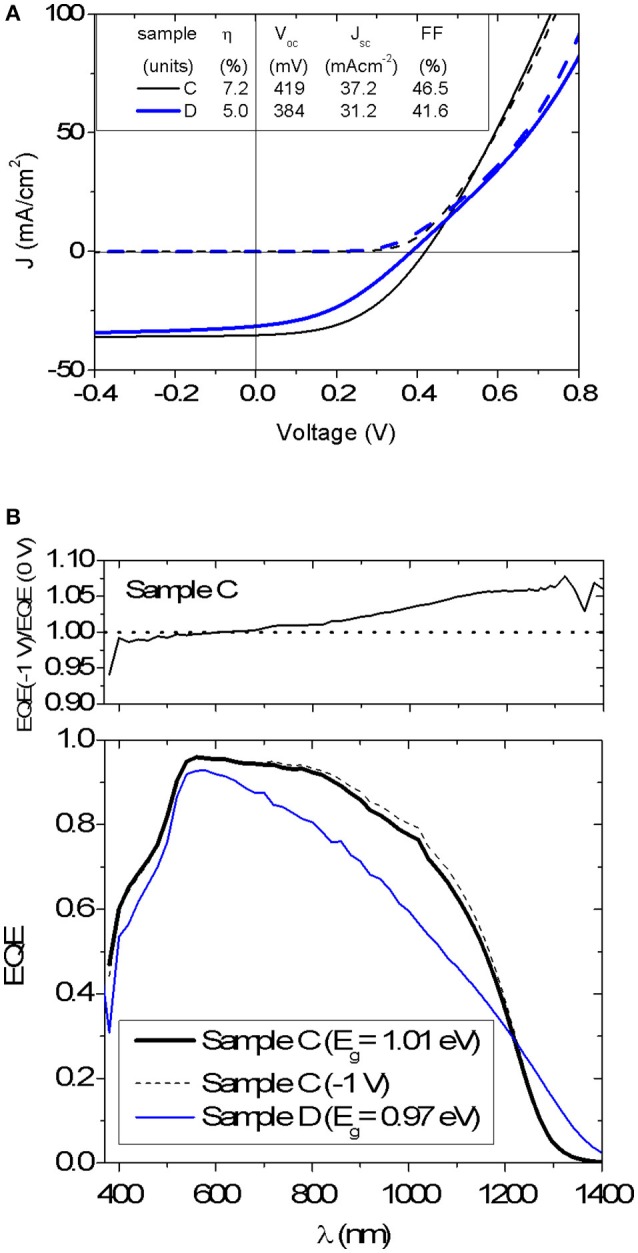
**(A)** J-V characteristic of the best devices C and D (dark curve—dash line, light curve—solid line). Photovoltaic parameters are also shown and refer to total area measurements. **(B)** External quantum efficiency of devices C and D. EQE spectrum for device C under a reversed bias of −1 V is also displayed as well as the ratio between voltage biased (−1 V) and unbiased EQE.

An average efficiency of 5.8%, V_oc_ = 405 mV, J_sc_ = 36.3 mA cm^−2^ and FF = 39.2% was measured for nine Mg-containing solar cells on sample C, whereas the nominally Mg-free sample D yielded an average efficiency of 4.2%, V_oc_ = 376 mV, J_sc_ = 29.0 mA cm^−2^ and FF = 38.2% for nine cells. J-V curves and EQE spectra for best cells from sample C and D are shown in Figure 6. It appears that the performance of Mg-containing cells is higher than that for nominally Mg-free devices, in particular by improving the charge collection in the long-wavelength part of the spectrum (Figure 6B). However, no reduction of the V_oc_ deficit is measured after adding Mg, being of 0.59 V for both best cells. The band gap energy E_g_ determined from the inflection point of the EQE spectrum is 1.01 eV and 0.97 eV for samples C and D, respectively. The comparison of reverse-biased EQE measurement with the EQE without bias is also plotted in Figure 6B for the device C. Although the Mg-containing device exhibits an enhanced carrier collection, it can be further improved under negative bias (−1 V). This bias dependence is signature of a poor carrier collection toward the back of the absorber layer (Scheer and Schock, 2011), which can be related to the smaller grain size next to the back contact.

**Figure 7:**
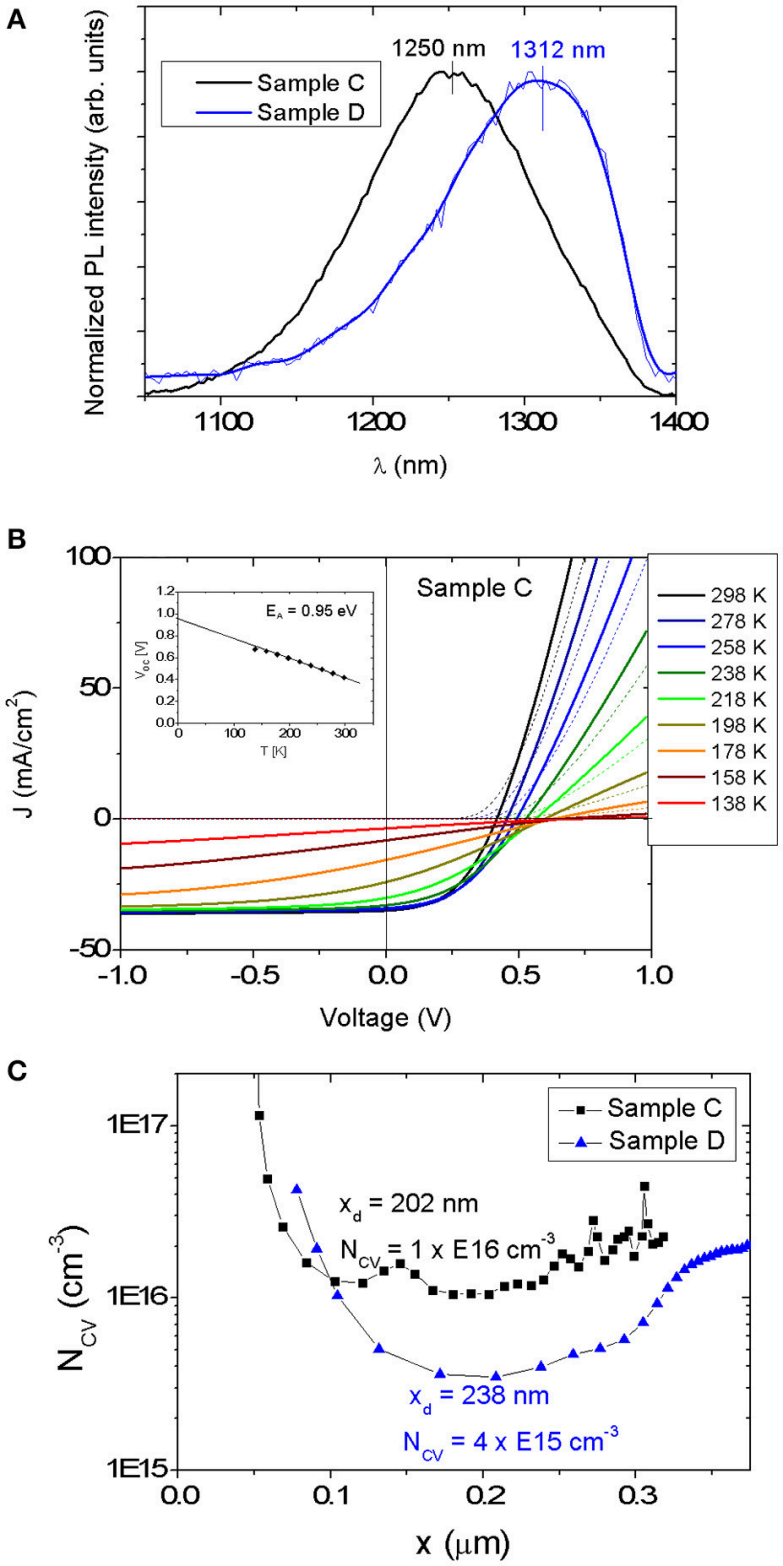
**(A)** PL spectra for samples C and D. The PL maxima occur at a smaller energy value than the band gap (as determined from the EQE curve) for both samples. **(B)** Temperature dependent J-V measurement (dark curve—dotted line, light curve—solid line) of device C. The inset shows a linear fit of V_oc_ that can be extrapolated to an intersection value E_A_ = 0.95 eV, which is close to the estimated E_g_ = 1.01 eV, as determined by EQE. **(C)** N_CV_ vs. x for the devices C and D extracted from C-V measurements. The values of depletion width x_d_ and N_CV_ at 0 V are shown.

Figure 7A shows PL spectra acquired at room temperature of devices C and D. The PL spectra exhibit a broad peak for both samples. The broad shape indicates the presence of tail states and/or potential fluctuations (Gokmen et al., 2013). Red-shifted PL peak maxima compared to E_g_, ΔE_Eg−PL_, by 20 meV and 25 meV are determined for solar cells C and D, respectively. These values are in the range obtained for CZTSe grown by vacuum-based methods (20 meV) (Lee et al., 2015).

Figure 7B displays the JV-T measurement of the device C. No roll-over effect is observed even at the lowest temperature of 138 K. The temperature dependence of the V_oc_ extrapolated to T = 0 K provides an activation energy E_A_ = 0.95 eV, which can correspond to the main recombination channel. Since this E_A_ value is very near the E_g_ of 1.01 eV determined by EQE, it appears that the dominant recombination path is located within the bulk of the kesterite absorber layer rather than at the CdS/CZTSSe interface.

Furthermore, capacitance-voltage (C-V) measurement was carried out for both solar cells (see Figure 7C). The depletion width (x_d_) of the Mg-containing CZTSSe device is approximately of 0.2 μm at a bias of 0 V. A carrier concentration N_CV_ of 1 × 10^16^ cm^−3^ can be extracted for the Mg-containing absorber, whereas a lower carrier concentration N_CV_ of 4 × 10^15^ cm^−3^ was measured for the Mg-free sample, indicating that the presence of Mg increases acceptor density. The doping level is in the same range as for Na-doped CZTSe solar cells fabricated using a NaF precursor layer and by diffusion from SLG substrates (x_d_ = 0.23 μm and N_CV_ = 1 × 10^16^ cm^−3^) (Lee et al., 2015).

## Conclusions

The incorporation of Mg into Cu_2_Zn_1−x_Mg_x_Sn(S,Se)_4_ layers leads to the complete phase separation for *x* = 0.5…1, whereas a lower content of *x* = 0.04 can preserve the kesterite phase. As compared to the nominally Mg-free sample, low quantities of Mg can improve the grain growth, reduce the number of structural defects and increase the acceptor density in the solar cell absorber. In this respect Mg appears to behave similar to the conventional alkali dopants such as Na or K. The first Mg-containing kesterite cell has been fabricated with the highest conversion efficiency of 7.2%. This value is lower than the efficiency of 11–12% obtained for Li, K or Na-doped devices (Haass et al., 2017), indicating that the addition of Mg is less effective.

## Author contributions

RC, SH, and YR designed the research and experiments. RC and SH fabricated solar cells, characterized layers and solar cells. CA assisted with the analysis. LA and FO carried out Raman spectroscopy measurements. VI-R assisted with Raman spectra analysis. RC, SH, FO, and YR wrote the paper. All authors contributed with discussions.

### Conflict of interest statement

The authors declare that the research was conducted in the absence of any commercial or financial relationships that could be construed as a potential conflict of interest.
