# Supplementary material for: Effect of Magnesium Incorporation on Solution-Processed Kesterite Solar Cells
Source: Front Chem. 2018 Jan 26;6:5. doi: 10.3389/fchem.2018.00005 (PMC5790964; doi:10.3389/fchem.2018.00005)
Supplement: Supplementary file 1 [file Table1.DOCX]

Supplementary Information- Table S1

Effect of Mg incorporation on Solution-Processed kesterite Solar Cells

Raquel Caballero*, Stefan G. Haass, Christian Andres, Laia Arques, Florian Oliva, Victor Izquierdo-Roca, Yaroslav E. Romanyuk

Table S1. Raman spectra parameters of samples C and D using 633 nm excitation wavelength. The values obtained are the average of measurements carried out on 9 cells of each type of samples.

| Sample | RS  Se-Se  [cm^-1^] | FWHM  Se-Se  [cm^-1^] | A(175)/  [A(196)+ A(175)] | A(242)/  [A(196)+A(242)] | [S]/  ([S]+[Se])  [%] |
| --- | --- | --- | --- | --- | --- |
| C | 196.2 ± 0.1 | 10.5 ± 0.3 | 0.272 ± 0.002 | 0.271 ± 0.003 | 4.6 ± 1.0 |
| D | 196.3 ± 0.2 | 10.3 ± 0.2 | 0.268 ± 0.002 | 0.270 ± 0.002 | 3.9 ± 1.0 |
