# Supplementary material for: Effect of Magnesium Incorporation on Solution-Processed Kesterite Solar Cells
Source: Front Chem. 2018 Jan 26;6:5. doi: 10.3389/fchem.2018.00005 (PMC5790964; doi:10.3389/fchem.2018.00005)
Supplement: Supplementary file 3 [file Table3.DOCX]

Supplementary Information- Table S3

Effect of Mg incorporation on Solution-Processed kesterite Solar Cells

Raquel Caballero*, Stefan G. Haass, Christian Andres, Laia Arques, Florian Oliva, Victor Izquierdo-Roca, Yaroslav E. Romanyuk

Table S3. Raman spectra parameters from the back of the absorber C after its mechanical lift-off.

| Sample C | RS  Se-Se  [cm^-1^] | FWHM  Se-Se  [cm^-1^] | A(175)/  [A(196)+ A(175)] | A(242)/  [A(196)+A(242)] | [S]/  ([S]+[Se])  [%] |
| --- | --- | --- | --- | --- | --- |
| Pos. 1 | 196.0 ± 0.1 | 11.1 ± 0.3 | 0.271 ± 0.002 | 0.277 ± 0.003 | 3.8 ± 1.0 |
| Pos. 2 | 195.5 ± 0.1 | 11.3 ± 0.3 | 0.271 ± 0.002 | 0.273 ± 0.003 | 4.7 ± 1.0 |
